# Sexual identity or religious freedom: could conversion therapy ever be morally permissible in limited urgent situations?

**DOI:** 10.1007/s40592-021-00132-6

**Published:** 2021-07-20

**Authors:** Owen M. Bradfield

**Affiliations:** 1 Law & Public Health Unit, Centre for Health Policy, Melbourne School of Population & Global Health, University of Melbourne, Parkville, Australia; 2 University of Oxford, Oxford, UK

**Keywords:** Conversion therapy, Heterosexism, Religious freedom, Sexual identity

## Abstract

Conversion therapy refers to a range of unscientific, discredited and harmful heterosexist practices that attempt to re-align an individual’s sexual orientation, usually from non-heterosexual to heterosexual. In Australia, the state of Victoria recently joined Queensland and the Australian Capital Territory in criminalising conversion therapy. Although many other jurisdictions have also introduced legislation banning conversion therapy, it persists in over 60 countries. Children are particularly vulnerable to the harmful effects of conversion therapy, which can include coercion, rejection, isolation and blame. However, if new biotechnologies create safe and effective conversion therapies, the question posed here is whether it would ever be morally permissible to use them. In addressing this question, we need to closely examine the individual’s circumstances and the prevailing social context in which conversion therapy is employed. I argue that, even in a sexually unjust world, conversion therapy may be morally permissible if it were the only safe and effective means of relieving intense anguish and dysphoria for the individual. The person providing the conversion therapy must be qualified, sufficiently independent from any religious organisation and must provide conversion therapy in a way that is positively affirming of the individual and their existing sexuality.

## Introduction

“Conversion therapy” (CT) refers to a range of discredited practices that attempt to re-align an individual’s sexual orientation, usually from non-heterosexual to heterosexual (Drescher et al. 2016). Also known as *reparative therapy*, *reorientation therapy* or *sexual orientation change efforts*, it can include psychotherapy, aversion therapy, exorcisms, spiritual cleansing, electric shock therapy or hormonal pharmacotherapy (Alempijevic et al. 2020). These practices are founded on unscientific and heterosexist assumptions that sexual orientation is mutable and that heterosexuality is socially, morally and legally favoured (Berg et al. 2016). “Homosexuality” was declassified as a mental disorder in 1973 (Asch 2009). This contributed to growing acceptance of homosexuality as a normal variant of human sexual expression. Many jurisdictions have now introduced legislation banning CT (Drescher et al. 2016). Despite this, CT persists in over 60 countries (Pérez-Sales 2020). The American Psychological Association (Asch 2009), the Australian Medical Association and the British Medical Association (Cohen 2010) now support affirmative care for non-heterosexual people and oppose efforts to change sexual orientation (Haldeman 2002). The American Psychiatric Association opposes “any psychiatric treatment… which is based upon the assumption that homosexuality per se is a mental disorder or…that the patient should change his/her homosexual orientation” (AMA 2000).

Much of the CT literature recognises that CT is unsafe, harmful and reinforces heterosexist norms. CT can lead to depression, suicide, self-loathing, shame, substance abuse, self-harm, social rejection and social isolation (Byne 2016). Children are particularly vulnerable to coercion, rejection, abandonment, blame and privacy breaches from their family (Berg et al. 2016), as well as school non-attendance and delayed psychosexual development (Turban et al. 2020). However, as our understanding of human sexuality increases, new biotechnologies such as neuropharmacology, brain surgery, deep brain stimulation, or genetic modifications might safely and effectively deliver sexual re-orientation in the future (Delmas and Aas 2018). In this paper, I will argue that the use of safe and effective CT could be morally permissible in very limited circumstances, such as when used to simultaneously relieve individual suffering and preserve family relationships. In addition, CT would need to be accessible, inexpensive, reversible and applicable to any sexual orientation (homosexual, heterosexual, bisexual, pansexual or asexual) as well as to children whose sexual orientation is emerging. I will analyse the arguments of Delmas and Aas (2018) and Earp et al (2014) in relation to the moral permissibility of CT in consenting adults and extend those arguments to children. I will mainly focus on children who are too young to consent to, but whose parents request, CT.

## The moral permissibility of conversion therapy in an ideal world

Queer theorists and LGBT rights activists rightly argue that CT is harmful because it perpetuates dominant heterosexist stereotypes. Delmas and Aas (2018) argue that, in an ideal world—one that has achieved full sexual equality and emancipation from heterosexism—having the option to alter one’s sexual orientation might be largely unnecessary because non-heterosexuality would not be viewed as inferior to heterosexuality. There would be no unjust discrimination or disadvantage from which a non-heterosexual person might want to escape.

In the case of children with an emerging non-heterosexual identity, CT might also be unnecessary for similar reasons. In the current sexually unjust world, parents seeking CT are motivated primarily by love and apprehension (Ryan and Rees 2012). They worry that, if their children identify as non-heterosexual, they will face stigma, discrimination, violence and social rejection (Ryan et al. 2009). Most parents want their children to be safe and treated fairly, although some may consider being non-heterosexual to be morally wrong in some way. However, in an ideal sexually just world, there would be no sexual prejudice or violence from which parents would seek to protect their children. Likewise, the extent to which children perceive emerging non-heterosexual desires with anxiety or distress will likely accord with the views of their parents, teachers, peers, community and wider society. If children receive clear messages that non-heterosexual orientation is good or normal, then children with emerging non-heterosexual desires will either not perceive themselves as different or will accept or value any perceived difference. CT would likely be anathema to a just society and it is unlikely that there would be any pressure or coercion imposed on children or their parents to extinguish or alter any particular emerging sexual orientation. Therefore, there would be no harm to individuals and minimal harm, if any, to sexual minority groups.

Delmas and Aas (2018) suggest that, despite being largely unnecessary in a sexually just world, the availability of CT may increase autonomy. For example, in a society where non-heterosexual desires and relationships are not disvalued, heterosexual individuals could conceivably choose to undergo CT in order to achieve a homosexual orientation. Close heterosexual friends of the same sex might want to develop a sexual and romantic relationship in order to more profoundly connect. Delmas and Aas (2018) argue that this increases individual autonomy without infringing upon the rights or freedoms of others. On this ground, Delmas and Aas (2018) believe that CT is morally permissible for adults in a just world. Indeed, others go further and argue that any technological intervention that expands one’s romantic attractions might be morally permissible (Thau 2020).

So how does this apply to children? What if 14-year-old Sally came home from school one day and told her parents that, in the future, she would like to marry her best friend Angela? How does a parent meaningfully interpret this comment? Is Sally expressing emerging homosexual desires? Is she expressing non-sexual affection for her friend? Or is she expressing a desire to change her emerging heterosexual orientation into a fixed non-heterosexual orientation? Or is she simply too young to understand the implications of her statement, which should be revisited once her sexual orientation has more fully emerged? If we offered Sally CT at the age of 14 years, would it threaten or enhance her authenticity?

Authenticity requires that moral agents make decisions that are not only autonomous, but are also cohesive with one’s core identity and higher-order values, by being true to oneself for one’s own benefit. The concept of authenticity has been used to argue against CT (Fjelstrom 2013). Members of the LGBTQI community describe ‘coming out’ as an affirmation of self-acceptance, bravery and authenticity, as opposed to ‘being in the closet’, which stifles their quest for authenticity (Fjelstrom 2013). Nonetheless, authenticity is a protean concept and determining an individual’s hierarchy of values is challenging, especially in children.

Children who lack mature cognitive capacities are likely to also lack a mature sense of “true self”. This makes it difficult to judge what decisions about sexual identity are consistent with the requirement to be authentic. Although children may have sexual intuitions, they may not fully recognise, understand or interpret them, particularly in a sexually just world where binary gendered concepts may not be recognised. It is therefore difficult to draw conclusions about the precise impact of CT on children’s authenticity or liberty, especially when their psychosexual identity is emerging. Moreover, in a sexually just society that may not tolerate attempts to shape a child’s psychosexual behaviours, arguments about enhancing children’s liberty though the use of safe and effective CT may be misplaced and largely redundant.

## The moral permissibility of conversion therapy in a non-ideal world

Stronger arguments have been made for rejecting CT in a non-ideal heterosexist world, even when CT is safe and effective. These arguments assert that CT perpetuates heterosexist oppression because it explicitly and implicitly pressures sexual minority groups to convert, by casting stigma, blame and responsibility on non-heterosexual people who choose not to (Delmas and Aas 2018). This reduces sexual diversity and collectively harms the identity and psyche of sexual minorities by demoting homosexuality from an innate and unassailable part of the self to a mere ‘lifestyle choice’ (Delmas and Aas 2018). Delmas and Aas (2018) conclude that, although the voluntary use of safe and effective CT could be beneficial to individuals in many circumstances, sexual orientation should remain outside an individual’s control because of the collective harms of CT to non-heterosexual people (Delmas and Aas 2018).

On the other hand, Earp et al (2014) argue that safe and effective CT could be allowed where it would benefit an individual, even if their request is motivated by internalized homophobic norms. In support of this argument, they cite the example of ultra-Orthodox Jewish Yeshiva students whose non-heterosexual desires and behaviours conflict with their religious teachings, leading to mental health problems and depression. In some cases, these students’ non-heterosexual behaviours are suppressed by psychiatrists using psychotropic medication at the request of rabbis and individual parents. Earp et al (2014) contend that, for individuals who deeply suffer on account of their homosexuality, and who are unable or unwilling to abandon the repressive norms that cause their distress, the genuinely voluntary use of CT should be morally permissible where it can resolve inner psychic conflict, relieve significant suffering and enhance autonomy. “The painful reality of individual suffering in the here-and-now presents a genuine predicament in need of some solution: how much personal well-being in today’s imperfect world must be sacrificed on the altar of future, society-wide progress in changing problematic social norms?” (Earp et al. 2014, p. 9). This is a compelling argument.

The Yeshiva student example lucidly portrays the power that religion exerts over individuals and parents and the harm that can be inflicted from discriminatory and heterosexist norms. Society has a moral obligation to oppose religious teachings that stigmatise non-heterosexual identities. Dawkins (2006) believes that children should be taught *how* to think, rather than being forced *what* to think. In his opinion, it is wrong for parents to decide the faith of their children and to label them as Christian or Muslim when a child cannot independently decide their faith. This view can be contrasted with that of Claudia Mills, who argues that it is impossible for parents to raise their children “without steering them, however imperceptibly, toward one option rather than another” (Mills 2003, p. 501).

However, even if society accepts that parents can and should choose the religion of their children, this does not entail that society should tolerate the compulsory inculcation of faith in ways that irreparably harm the psychological well-being of children who must endure a childhood dominated by propaganda and fear of disapproval or eternal damnation.

If your whole upbringing, and everything you have ever been told by parents, teachers and priests, has led you to completely believe that sinners burn in hell, it is entirely plausible that words could have a … long-lasting and damaging effect ... I am persuaded that the phrase 'child abuse' is no exaggeration when used to describe what teachers and priests are doing to children whom they encourage to believe in something like the punishment of unshriven mortal sins in an eternal hell (Dawkins 2006, p. 318).

By analogy, the traumatisation of children resulting from the systematic indoctrination of the belief that non-heterosexual attraction is an abomination could also amount to child abuse. If this is the case, then society must protect these children from the anticipated and intolerable suffering and alienation that ensues.

There are several ways in which society might attempt to avert or abrogate such harm. One is to prevent religious organisations and parents from conspiring to misinform children about the true nature of non-heterosexual feelings. However, powerful religious organisations vehemently resist attempts to regulate their conduct and affairs. In many countries, they enjoy exemption from anti-discrimination laws, where the discriminatory act or conduct otherwise conforms to the “doctrines, tenets or beliefs of the religion, or is necessary to avoid injury to the religious sensitivities of adherents of that religion”.^1^ Similar laws exist across our sexually unjust world. Insofar as the state affords special status to religious institutions and specifically sanctions discriminatory practices, it seems unlikely that discriminatory religious education could be effectively proscribed in a liberal democracy in the foreseeable future.

Another way of preventing harm might be to re-educate children to help them understand that non-heterosexual feelings are a normal variant of sexual expression. This could be challenging when a child has been taught that truth comes from scripture, rather than from evidence. Parents might refuse to consent to such re-education. It is therefore unclear how or where this could be realistically achieved. If a re-educated child too openly questions homophobic or discriminatory religious norms and teachings, they may be rejected by their parents or religious leaders and forced to either repent or abandon a belief-system that binds their family, friends, community and culture. We need to be cautious not to coercively regulate religious education, as this might replace religious indoctrination with political indoctrination. For example, the “re-education” of Uyghur minority Muslims in the Xinjiang Province of China demonstrates the consequences of excessive government intercession to combat “religious extremism” through systematic censorship and forced renunciation of religion and culture (Xu 2020). After all, who decides the “truth” of what religions can and cannot teach?

Since the establishment of the New York Society for the Prevention of Cruelty to Children in 1874, most governments have created services with legally enforceable mandates that protect children from abusive parents or caregivers (Higgins 2011). Therefore, a more radical option might be to remove children from families where emerging non-heterosexual feelings put them at significant risk of psychological harm from homophobic religious teachings. However, we know from the experience of Australia’s Stolen Generations^2^ that removing children from their families and communities can break important spiritual bonds and can have pervasive and intergenerational effects (Wilkie 1997). Research overwhelmingly shows that children disconnected from their cultural identity and heritage have higher rates of mental illness and criminal offending and lower levels of education and employment (Bushell and Stevens 2000). Clearly, reasonable effort should be made to keep children within their family and community networks to the extent possible.

So, in the absence of viable alternatives, safe and effective CT could be cautiously adopted in limited situations where it relieves the child’s extant suffering, prevents social dislocation, preserves their cultural identity and strengthens family attachments. However, one important criticism of this view is that CT indirectly harms sexual minority groups. Using the Yeshiva student example, it is clear that religious organisations and health professionals have frequently colluded in promoting CT to reinforce their dogma: psychiatrists offer CT to Yeshiva students at the behest of rabbis. Likewise, in the US, organisations offering CT often employ counsellors sympathetic to the organisation’s philosophies (Will et al. 2018).

Health professionals must always put the interests of their patients above their own. This is the foundation of trust that underpins the practitioner-patient relationship. If a practitioner offering CT is also mired in, and subservient to, heterosexist religious dogma, they may be unable to exercise impartial clinical judgment. Consequently, they may be unwilling to recognise the harm caused by heterosexism and unable to recognise that CT may compound that harm. In a sexually unjust world where vulnerable children who are victims of institutionalised homophobia are seeking advice and treatment, it is crucial that safe CT is also discussed and delivered in a culturally safe space and manner by competent and independent practitioners. Ideally, CT should not condone harmful religious teachings. Instead, it should focus on relieving distress by positively affirming the child’s individual choice and existing sexuality. Unfortunately, the practical reality is that this may be difficult to achieve and many practitioners may be influenced by religious or social biases that impair their ability to provide objective, safe and effective care. This is ultimately problematic for any conclusion that recommends CT in all but the most serious or urgent of situations.

Another argument against the use of CT to repair psychologically damaged children is that those children may become future parents who perpetuate religious dogma and harm on their own children: this argument holds that CT facilitates, rather than rejects, religious vilification. Nevertheless, the counter-argument to this view is that the urgent need to relieve the extant and tangible suffering of a child today does not obviate the need to continue to rally against religious practices that are detrimental to the emotional and psychological well-being of children tomorrow. Society has a moral obligation to do both. It must simultaneously relieve, prevent and reduce suffering. By analogy, treating injured soldiers allows them to return to battle, but does not subvert or obviate the need to decry war and seek peace. Likewise, welcoming refugees from countries ravaged by homophobic persecution is not evidence of our acceptance of the homophobic persecution from which they seek refuge. Instead, we are moved to intervene through compassion for the profound suffering of the individual. So it is that CT could be morally permissible in very limited circumstances.

## Conclusion

CT generally causes significant and long-term harm, largely because of a lack of proven safety or efficacy. However, even if its safety and effectiveness could be assured, it perpetuates heterosexist and homophobic norms that can injure sexual minority groups. In this paper, I have argued that, in an ideal sexually-just world, safe and effective CT may be morally permissible, but would probably be unnecessary. However, in the current non-ideal world, caution is required. The example of ultra-Orthodox Jewish Yeshiva students demonstrates that CT could be morally justifiable only in very limited circumstances, such as where non-heterosexual desires are causing significant distress and acute risks to a person’s life. However, the practitioner offering the CT must be independent and unbiased, and other options must be either unavailable or ineffective. The practical reality is that these requirements may be difficult to achieve. Nonetheless, society has a moral duty to at least consider the role of CT in rescuing children from intolerable suffering and distress caused by fear of eternal damnation or rejection. Simultaneously, society has an obligation to resist and temper the underlying religious norms and teachings that precipitate these harms. Thus, while legislation banning CT must surely be applauded, it does not reach the root causes of sexual intolerance and discrimination that creates a sexually-unjust world, and an industry in discredited CT practices.

## Footnotes

1 *Sex Discrimination Act 1984* (Cth), ss 23(3)(b), 37 and 38.

2 This refers to the children of Aboriginal and Torres Strait Islander descent who were removed from their families by Australian governments, churches and welfare organisations between 1905 and 1967.
